# Prevalence of *pfhrp2/pfhrp3* gene deletions among patients with *Plasmodium falciparum* malaria with false-negative in the HRP2-based rapid diagnostic test in Colombia

**DOI:** 10.1590/0074-02760240134

**Published:** 2025-06-13

**Authors:** Mario Javier Olivera, Angela Patricia Guerra, Liliana Jazmín Cortés, Aravy Geohanna Suárez-Jurado, María de la Paz Ade, Iván Mauricio Cárdenas

**Affiliations:** 1 Instituto Nacional de Salud, Grupo de Parasitología, Bogotá DC, Colombia; 2 Pan-American Health Organization, Department of Communicable Diseases Prevention, Control and Elimination, District of Columbia, Washington, United States; 3 Ministerio de Salud y Protección Social, Subdirección de Enfermedades Transmisibles, Bogotá DC, Colombia

**Keywords:** Colombia, rapid diagnostic test, deletion, HRP2, Plasmodium falciparum

## Abstract

**BACKGROUND:**

In malaria-endemic regions, rapid diagnostic tests (RDTs) play a crucial role in promptly identifying infections, especially in remote areas with limited microscopy services.

**OBJECTIVES:**

Conduct a cross-sectional, multi-site study to determine whether the local prevalence of mutations in the *Plasmodium falciparum hrp2/3* genes in false-negative RDTs has reached a threshold that might require a local or national change in diagnostic strategy in accordance with the WHO guidelines (2018).

**METHODS:**

Individuals were screened for *P. falciparum* with microscopy and HRP2-based RDT at health facilities. Discordant results between these two tests triggered diagnostic confirmation by polymerase chain reaction (PCR) and detection of the *pfhrp2/pfhrp3* genes.

**FINDINGS:**

Among the 347 patients included, false negatives constituted 4.61% (16/347). Molecular analysis revealed all 16 false negatives were *P. falciparum* positive with *hrp2* gene present, displaying high polymorphism. However, *hrp3* gene deletion was observed in 93.8% (15/16) of these cases.

**MAIN CONCLUSIONS:**

The prevalence of false-negative RDTs is low, and these results were not linked to deletions in the *hrp2* gene. This suggests that there is no immediate need to modify the RDTs used along the Colombian Pacific Coast. However, molecular surveillance for *hrp2* deletions remains crucial to detect any potential increase in prevalence.

As the Americas progress towards malaria elimination, access to high-quality and timely diagnosis remains a challenge in high burden areas. Parasitological diagnosis is the fundamental basis for treatment, surveillance, and response, helping to prevent complications and fatal outcomes while reducing the costs associated with disease management and care.^1^

According to the World Health Organization (WHO), an estimated 263 million cases of malaria occurred worldwide in 2023, resulting in 597,000 deaths. These statistics highlight the significant impact of the disease on global public health and the ongoing need for prevention, detection and effective treatment of malaria. The African region accounted for 95% of the global malaria burden, concentrated in 29 countries. In the Americas, 72% of the regional malaria burden was recorded in the Bolivarian Republic of Venezuela, Brazil, and Colombia.^2^

In Colombia, malaria remains a significant public health concern. In 2023, approximately 105,479 cases were reported, of which 37,851 (35.8%) were attributed to *Plasmodium falciparum*. Furthermore, 84.8% (32,116) of these cases were concentrated in the Pacific region.^3^ To address this challenge, national malaria elimination strategies promote systematic actions for detection, diagnosis, and response. It is crucial to implement these strategies on a large scale and monitor them systematically to ensure effective diagnostic testing and appropriate treatment.^4^

Rapid diagnostic tests (RDTs) are useful tools for the timely diagnosis of malaria, when microscopy is unavailable, particularly in remote rural settings where access to health services is limited. These tests detect various antigens, including histidine-rich protein 2 (HRP2)^5^ and the enzymes lactate dehydrogenase and aldolase.^6^ HRP2 is a specific protein of *P. falciparum* and is the main target of the most widely used RDTs today. However, some strains have deletions in the genes encoding the HRP2 and HRP3 proteins, implying that these strains cannot be detected by the HRP2-targeting RDTs.^7^

The first evidence of parasites carrying deletions in the *pfhrp2* and *pfhrp3* genes was reported in 2010 in South America, specifically in the Amazon River basin in Peru.^8^ Since then, various studies have documented these genetic deletions in several African countries, showing frequencies ranging from 0.4% in Angola, 10.7% in Zambia, 23% in Rwanda, 36.2% in Ghana, to 62% in Eritrea.^9^ ^,^ ^10^ In Asia, reports from India indicate deletion rates ranging from 2.4%.^11^ In South America, Brazil reported a deletion rate of 100% in Manaus, Amazonas State,^12^ while Peru documented a rate of 61% in Iquitos, located in the Peruvian Amazon region.^8^ In Colombia, the presence of parasites lacking the *pfhrp2* and *pfhrp3* genes has also been identified in regions such as the Amazon and in municipalities like Tierralta, Tumaco, and Buenaventura.^13^ However, these findings come from studies that did not focus on false-negative results from RDTs for *P. falciparum* and do not follow the WHO protocol.^14^ To date, there are no available data from Colombia following this protocol. This clarification is important for contextualizing the findings and the methodology used in previous studies. Importantly, in areas with a high prevalence of *pfhrp2* gene deletion, it may be necessary to use RDTs based on antigens other than HRP2 to ensure adequate detection of *P. falciparum* infection.^7^ According to the guidelines set by the WHO, a prevalence of *hrp2* deletions exceeding 5% necessitates a transition from HRP2-based RDTs to an alternative testing method.^14^ ^,^ ^15^

In settings where microscopy is not available or feasible due to staff or resource limitations, it is essential that malaria be treated according to the results of the RDTs; therefore, ensuring the accuracy of these tests is essential. RDT performance can be affected by various factors, such as product quality and yield, transport, or storage conditions, human error during the use of the test, the presence of a parasite density below the detection limit, or, as already mentioned, the absence of the genes that encode the HRP2 antigen.^6^ ^,^ ^7^ ^,^ ^16^ All these factors can contribute to false-negative results.

Given the crucial importance of HRP2 detection in malaria diagnostic strategies, the WHO is urging countries at risk of malaria transmission to assess the prevalence of deletions of the *pfhrp2* and *pfhrp3* genes in *P. falciparum*. This approach will ensure the effectiveness of RDTs and improve the accuracy of malaria diagnosis in places where microscopy is not a viable option. In this context, the present study aimed to measure the prevalence of suspected false-negative HRP2 RDT results among patients from Pacific Coast attending public health facilities with *P. falciparum* infection detected by microscopy and to determine the prevalence of deletion of the *pfhrp2* and *pfhrp3* genes in false-negative results in the RDTs.

## SUBJECTS AND METHODS

*Study design* - An observational cross-sectional multi-site study was carried out in people who sought medical attention at health facilities due to suspected malaria. The study design followed the WHO guidelines published in 2018 for estimating the prevalence of *pfhrp2/pfhrp3* gene deletions among falciparum malaria patients with false-negative RDT results.^14^ Microscopy and an HRP2-based RDT were used as diagnostic tests.

**Fig. 1:**
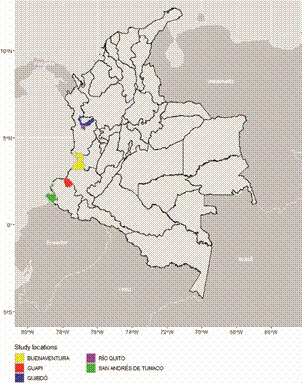
map of Colombia showing the sites where was carried out the study.

*Study sites* - The study was carried out in five malaria-endemic municipalities that are located along the Colombian Pacific coast: Quibdo-Choco, Rio Quito-Choco, Guapi-Cauca, Buenaventura-Valle, and Tumaco-Nariño (Fig. 1). The samples came from ten sentinel sites: four in Quibdo, one in Rio Quito, two in Guapi, one in Buenaventura, and two in Tumaco. The area is characterized by a tropical climate as well as a mountainous geography with partially forested areas. The main economy of the area is based on industrial deep-sea fishing, mariculture (cultivation of marine organisms for food products), forestry extraction, industrial gold and platinum mining, livestock, and agriculture (mainly African palm, banana, and plantain).

In Colombia, malaria is transmitted in an endemic-epidemic manner, resulting in an unstable transmission pattern with four macro-foci widespread across the national territory: Pacific Coast, Uraba-Bajo Cauca-Alto Sinu, Amazon-Orinoco, and Colombia-Venezuela border. In 2023, the reported malaria cases in the sentinel municipalities were as follows: Quibdo-Choco: 6,978; Rio Quito-Choco: 2,108; Guapi-Cauca: 2,934; Buenaventura-Valle: 1,714; and Tumaco-Nariño: 1,515.^3^

*Sample size* - Given the limited information on the prevalence of false-negative results in HRP2-based RDTs and its association with the deletion of the *pfhrp2* gene, an initial sample size of 370 patients with *falciparum* malaria was established following WHO protocol.^14^ Thirty-seven patients were included at each sentinel site.

This sample size was considered adequate to demonstrate, with 95% confidence, that the prevalence of suspected false-negative HRP2 RDT results and *pfhrp2/3* gene deletions at the sampling domain (*e.g.*, municipal level), was less than the threshold of 5%, for an expected prevalence in the population of 3.2%.

*Patients and procedures* - Between November 2020 and August 2021, adult participants (18 to 65 years old) who required medical assistance due to suspected malaria were recruited from ten health facilities.

Only patients with a confirmed diagnosis of *P. falciparum* by microscopy and parasitemia greater than or equal to 200 parasites/µL were enrolled. These patients were informed about the objectives of the study and were invited to participate. Individuals with *P. vivax* or other non-*P. falciparum* malaria, including mixed infections, and those with signs and symptoms of severe malaria were excluded from the study, receiving corresponding attention in each health facility as per the national guidelines.

The aims and methods of the study were clearly explained in Spanish, and every eventual participant signed informed consent. Blood was drawn from a second finger prick to prepare two thick smears, perform an RDT, and impregnate 100 µL of blood on two filter-paper circles for molecular testing if necessary. The filter-paper cards were air-dried for 24 h and stored in resealable bags containing a silica gel pad, while the thick blood smears were stained with Field’s stain before transferring them to the Parasitology Laboratory of the National Institute of Health for analysis.

After sample collection, patients received treatment for malaria according to the national guidelines of the Ministry of Health of Colombia and were asked to complete a questionnaire included in the study protocol.

*Microscopy* - Thick blood smears were prepared from capillary punctures and stained according to National Reference Laboratory standards.^17^ Expert microscopists diagnosed malaria, specifically *P. falciparum*, using morphological keys. Parasite counts were calculated using the formula (number of parasites × 8000) / number of leucocytes (200 or 500, as appropriate). Positivity, species identification and parasite count in parasite units/µL of blood were evaluated on each slide. All slides were subjected to quality control by the Parasitology Group to verify the results.

*RDT* - The RDT used was a rapid, qualitative test to detect *P. falciparum* HRP2 antigen and *P. vivax* pLDH in whole blood. It has a sensitivity of 99.7% and specificity of 99.5% for *P. falciparum*, and a sensitivity of 95.5% and specificity of 99.5% for *P. vivax*. This WHO-prequalified RDT requires a 5 μL blood sample and provides results in 15 min.

The test was performed by capillary puncture, following the manufacturer’s instructions. After applying the blood and buffer, the cassette was left in a horizontal position for 15 min. The results were marked, and all cassettes were sent to the Parasitology Group for quality control of positivity and species identification.

*Polymerase chain reaction (PCR) for Plasmodium detection* - Discordant samples between microscopy and RDT results were processed using nested PCR to confirm *P. falciparum* monoinfection, targeting the 18S ribosomal RNA gene following established protocols.^18^ ^,^ ^19^

*Deletion of the pfhrp2 and pfhrp3 genes* - Confirmed *P. falciparum* samples underwent semi-nested PCR to detect exon 2 of the *hrp2* and *hrp3* genes, by using primers from Baker et al.^20^ Agarose gel electrophoresis was used to resolve the PCR products, with expected amplicon sizes around 600-1200 bp. If no amplification of *pfhrp2/3* genes occurred, samples were tested for amplifiable DNA using nested PCR for *pfmsp1*.^21^

*pfhrp2 gene sequencing and bioinformatics analysis* - PCR products of the *pfhrp2* gene were sequenced using the Sanger method. The consensus sequence was generated with the EMBOSS Cons tool, and translated into amino acids using the ExPASy Translate tool.^22^ Amino acid repetitions were quantified, and sample sensitivity was categorized based on the type 2 × type 7 score.^23^

*Definitions and statistical analysis* - For this purpose, from the daily record format, a database was built in MS Excel (Microsoft, Redmond, USA), where all the variables were recorded, and quality control of the data was performed. False-negative results were defined as samples with a negative result for RDT and positive microscopy. The prevalence of false-negative HRP2-based RDT results was established by dividing the total number of discordant samples (microscopy-positive and RDT-negative samples) by the total number of samples positive for *P. falciparum* by microscopy, both locally and nationally. The prevalence of false negatives in HRP2-based RDTs caused by pfhrp2/3 deletions was determined by dividing the number of samples with *pfhrp2* or *pfhrp3* deletion by the total number of discordant samples. The sensitivity for HRP2-based RDTs of the discordant samples was predicted by calculating the number of repeats present in HRP2, specifically the score between type 2 and type 7 repeats. The value between type 2 × type 7 classifies the samples into the following categories: a (very sensitive, > 100), B (sensitive, 50-99) and C (low/non-sensitive, < 50).

For statistical analysis, Stata version 14.0 (Stata Corporation LP, College Station, TX, USA) was used. The measures of central tendency and dispersion that were applied included the geometric mean with 95% confidence interval (CI) for continuous variables and the absolute frequency and percentage for the categorical variables. The study also included measures of relative frequency, including general malariometric indices, such as the annual parasite index (API), which is defined as the number of confirmed cases of malaria per 1,000 inhabitants at risk.

*Ethical approval and consent to participate* - Ethical approval was obtained from the Research Ethics and Methodologies Committee of the National Institute of Health in Bogotá, Colombia (CEMIN 44-2019) and the Pan-American Health Organization Ethical Review Committee (PAHOERC 0233.01). All included patients gave their voluntary consent to participate.

## RESULTS

*General findings* - Three hundred and seventy patients with *P. falciparum* infection were initially enrolled in the study. After quality control of the thick blood smears and the RDTs at the National Institute of Health, 23 participants who did not meet the inclusion criteria were excluded^,^ so that the data analysis was performed on 347 patients. The baseline characteristics of participants are shown in Table I. The mean age was 34.5 years (with a standard deviation of 12.9 years and a range of 18-65 years), and 184 of them (53%) were men. The geometric mean of parasitemia was 3,624 parasites/μL of blood (95% CI: 3,072-4,274). The patients who were excluded from the sample had similar characteristics to the subjects included in the analysis.

**TABLE I:** Baseline characteristics of the 347 patients included in the study

Variable	Buenaventura	Quibdo				Rio Quito	Guapi		Tumaco	
	SS1	SS2	SS3	SS4	SS5	SS6	SS7	SS8	SS9	SS10
API	5	52			244	103	6
Sex										
Female	20 (54%)	14 (41%)	16 (43%)	12 (32%)	13 (35%)	20 (69%)	15 (50%)	12 (33%)	25 (68%)	24 (73%)
Male	17 (46%)	20 (59%)	21 (57%)	25 (68%)	24 (65%)	9 (31%)	15 (50%)	24 (67%)	12 (32%)	9 (27%)
Age (y), median (IQR)	33.8 (19-64)	29.7 (18-65)	33.4 (18-65)	32.2 (18-65)	33.6 (18-62)	29.6 (18-56)	34.3 (18-65)	30.7 (18-60)	31.0 (18-62)	35.2 (18-64)
Self-administered antimalarials in last 15 days										
Chloroquine	2 (5%)	0 (0)	0 (0)	1 (3%)	0 (0)	0 (0)	0 (0)	0 (0)	0 (0)	0 (0)
Travel history in last 30 days										
Endemic malaria zone	20 (54%)	14 (41%)	19 (51%)	15 (41%)	25 (68%)	0 (0)	0 (0)	13 (36%)	10 (27%)	0 (0)
RDT result										
False-negative for *Plasmodium falciparum*	0 (0)	6 (18%)	3 (8%)	3 (8%)	3 (8%)	0 (0)	0 (0)	1 (3%)	0 (0)	0 (0)
Microscopical result										
*P. falciparum*	37 (100%)	34 (100%)	37 (100%)	37 (100%)	37 (100%)	29 (100%)	30 (100%)	36 (100%)	37 (100%)	33 (100%)
Geometric mean parasite (parasites/μL)	3,600	2,614	3,028	3,125	3,395	12,646	2,326	3,335	5,702	4,032

API: annual parasite index x 1,000 inhabitants; SS: sentinel site; y: Years, IQR: Interquartil.

*Prevalence of false negative results in HRP2-based Malaria RDT* - A prevalence of false negatives in HRP2-based RDTs of 4.6% (16/347) was detected among patients with a positive diagnosis of *P. falciparum* by microscopy. Of these, 0.28% (1/347) occurred in the municipality of Guapi-Cauca, and 4.32% (15/347) occurred in the municipality of Quibdo-Choco. No false negatives were identified in the samples obtained from the other municipalities, *i.e.*, Buenaventura, Tumaco, and Rio Quito.

False-negative results were seen in 81.2% (13 slides) of the patients with parasitemia of less than 1,000 parasites/μL. However, the remaining 18.8% (three slides) had a parasitemia greater than 1,000 parasites/μL, varying between 2,509 and 14,414 parasites/μL, and these did not show positive reaction bands for *P. falciparum* on RDT.

**Fig. 2:**
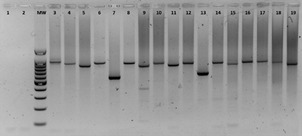
agarose gel electrophoresis displaying the semi-nested polymerase chain reaction (PCR) products of the amplification of *pfhrp2* exon 2. Lanes 1 and 2 correspond to negative controls with water and DNA from Haiti strain lacking the *pfhrp2* gene. Lanes 3 to 18 are clinical samples showing a PCR product between 1000 and 1200 bp as expected. Samples QB33 (Lane 7) and QT37 (Lane 13) exhibit a smaller size compared to other samples, indicating a partial deletion of *pfhrp2* exon 2. DNA from 3D7 strain was used as amplification control (Lane19). MW: 100 bp DNA Ladder, Promega. In the marker lane, the bands corresponding to 1500, 1000, and 500 bp are shown.

*Detection of the pfhrp2 and pfhrp3 genes in patients with a positive thick film for P. falciparum and negative RDT* - Through the molecular analysis of the 16 samples that were diagnosed as positive for *P. falciparum* in the thick film but did not present a positive reaction band for *P. falciparum* in the RDT, we found that 100% of the samples (16/16) were positive for *P. falciparum* and that the *pfhrp2* gene was present. However, the size observed in the amplification of two of the 16 samples evaluated by PCR showed a partial deletion, which was detected as a reduction of approximately 100 bp from the expected size (Fig. 2). The results for the *pfhrp3* gene were completely different, since the gene was detected in only one sample (Guapi), while it was absent in 15 of the discordant samples [Supplementary data (Figure)]. The total prevalence of *hrp3* gene deletion was 93.75% (15/16).

*Variation in the HRP2 sequences from false-negative RDTs* - Fourteen of the 16 samples evaluated were selected based on the quality of the sequences determined by the review of the chromatograms. Bioinformatics analysis of the 14 sequences showed that they maintained the repetition pattern described in other HRP2 genotyping studies in different areas of the world,^24^ ^,^ ^25^ ^,^ ^26^ the most frequent repetitions being type 2 (AHHAHHAAD) and type 7 (AHHAAD), with an average of 10 and five repetitions per sample, respectively. The calculation of the type 2 × type 7 score classified nine samples as sensitive and six as non-sensitive (Table II), following the method of Thang et al.^23^

**TABLE II:** Categorization of sensitivity to rapid diagnostic test (RDT) based on the type 2 (AHHAHHAAD) x type 7 (AHHAAD) HRP2 repeats present in each sample

Sample	Type 2	Type 7	Score	Category^*a*^	Sensitivity	Parasitemia	*pfHRP3* gene
QB17	12	5	60	B	Sensitive	216	Absent
QB18	10	8	80	B	Sensitive	381	Absent
QB24	11	6	66	B	Sensitive	692	Absent
QB33	3	4	12	C	Low/No sensitivity	212	Absent
QB36	8	6	48	C	Low/No sensitivity	915	Absent
QH11	10	7	70	B	Sensitive	320	Absent
QH26	11	7	77	B	Sensitive	14,414	Absent
QT14	10	7	70	B	Sensitive	212	Absent
QT36	12	5	60	B	Sensitive	429	Absent
QT37	10	2	20	C	Low/No sensitivity	579	Absent
QT57	8	4	32	C	Low/No sensitivity	4,000	Absent
QH20	9	5	45	C	Low/No sensitivity	302	Absent
QT70	14	5	70	B	Sensitive	248	Absent
GA12	12	4	48	C	Low/No sensitivity	2,509	Present
3D7^*b*^	11	5	55	B	Sensitive	-	Present

*a*: the categories shown are type A (score >100, “very sensitive”), type B (score 50-99; “sensitive”) or type C (< 50, “low/no sensitivity”); *b*: reference strain.

## DISCUSSION

The present study highlights four important findings in the main malaria-endemic areas on the Colombian Pacific Coast in *P. falciparum*-positive patients. First, no parasites with deletion of the *hrp2* gene were found in samples with discordant result between microscopy and RDT. This is significant since the presence of this deletion affects the performance of the RDTs and the timely diagnosis of the disease. Second, the frequency of *hrp3* gene deletion was high. This finding might cause a decrease in sensitivity of RDT due to the lack of expression of the HRP3 protein. Third, a low prevalence of false-negative results was observed in the RDTs. These findings indicate that the tests used are reliable and provide accurate results in the detection of malaria in most cases. Fourth, the false-negative results for the RDTs were not associated with deletion of the *hrp2* gene, which indicates that the HRP2-based RDTs currently utilised in the country can continue to be used in the endemic areas studied since they are highly effective at detecting malaria and can be implemented in areas where microscopy is not available. Therefore, these RDTs continue to be a very useful tools for the prompt diagnosis and treatment in this endemic region.

In general, the results of the present study agree with those reported in Ecuador, where in 41 samples collected in the provinces of Esmeraldas and Carchi between 2019 and 2020, no deletion was found in the *pfhrp2* gene, but the *pfhrp3* gene was deleted in 22 of the 41 samples (53.7%).^26^ In contrast, neighboring countries such as Brazil and Peru have reported a high prevalence of both deletions, especially in parasites from the Amazon basin.^12^ ^,^ ^27^ In Peru, a prevalence of 71% was reported in 324 samples collected between 2011 and 2018.^27^ ^,^ ^28^ Other investigations in the Brazilian Amazon region have confirmed the presence of parasite populations with high frequencies of deletion of the *pfhrp2* gene, as observed in the states of Amazonas 100% (60/60) and Acre 71.7% (71/99).^12^ Colombia is no exception to this situation, as indicated by the high prevalence of *P. falciparum* parasites lacking the *pfhrp2* gene in the Amazon region, thus, one study reported a prevalence of deletion of the *pfhrp2* gene of 38.5% (15/39)^29^ and another of 77.8% (14/18).^13^ Our findings together with the lack of the *hrp2* gene in Amazon parasites suggest that different genetic variants of the parasites circulate in Colombia.

In relation to the *pfhrp2* gene, the results obtained in this study contrast with those reported in investigations carried out in three municipalities on the Colombian Pacific coast, which were also covered by the present study. In Guapi (Cauca) a 6.2% (2/31) deletion in the *pfhrp2* gene was found in samples collected between 2014 and 2017.^30^ Likewise, in Tumaco (Nariño) and Buenaventura (Valle del Cauca), deletion rates of 2.9% (1/34) and 4% (1/25), respectively, were reported in historical samples collected between 2008 and 2009. On the other hand, in the municipality of Tierralta (Córdoba), located in the Caribbean region of the country, a deletion prevalence of 12.5% (2/16) was reported. The design of these studies is completely different from ours, as our work was carried out in the context of false-negative HRP2-based RDTs, and this could partly explain the differences in the prevalence values.

Our results showed a high prevalence of deletion of the *pfhrp3* gene in false-negative RDTs in Quibdo. The deletion of *pfhrp3* has also been observed in other of the malaria areas of Colombia and in some countries of the Americas. However, in Peru, the deletion of the *pfhrp2* gene predominates (71%) over that of the *pfhrp3* gene (67%),^27^ ^,^ ^28^ as well as in Suriname, where there has been a higher prevalence of *pfhrp2* deletion than of *pfhrp3* deletion.^31^ In general terms, in the region of the Americas, there is a very high prevalence of deletion of the *pfhrp3* gene. It is important to keep this scenario in mind, as some monoclonal antibodies against HRP2 present in RDTs cross-react with HRP3 due to the numerous repetitive epitopes conserved between both proteins.^32^ Consequently, when these RDTs are run on samples that contain parasites with deletion of the *hrp2* gene, but not the *hrp3* gene, they can generate positive results.^33^

During the present study, an acceptable prevalence (4.6%) of false-negative results was observed in the region with the highest malaria burden in the country; however, when reviewing by locality, the prevalence of false-negative results was higher in Quibdo than in Guapi, and it was not associated with deletion of the *pfhrp2* gene. The discrepancy between the results of microscopic diagnosis and the RDT is worrying, as it can lead to a misdiagnosis of malaria infections. Various causes may explain these results. First, the density of parasites may be below the detection limit of the RDT, which is typically within the range of 200 parasites/μL. Kong and colleagues showed that in culture samples with parasite densities of approximately 1,000 parasites/μL, the antigen concentration was much higher, when the *hrp2* and *hrp3* genes were intact (strain 3D7: hrp2+/hrp3+ = 47.9 ng/mL), than it was in strains where one of the two genes was absent (HB3: *hrp2*+/*hrp3*- = 3.02 ng/mL; and Dd2: *hrp2*-/*hrp3*+ = 0.20 ng/mL). At a density of 10 parasites/μL, only the strain with two intact genes (0.45 ng/mL) was reactive in the RDT, while neither of the other parasites was reactive at that density.^34^

In order to understand the false-negative results in RDTs, the calculation of the type 2 × type 7 score was carried out in the discordant samples. Six of the samples analyzed (37.5%) were classified in the non-sensitive category due to the lower frequency of said repetitions, which was evidenced by the smaller size of the amplicons. Baker et al. and Lee et al.^20^ ^,^ ^35^ previously established a relationship between the number of type 2 and type 7 repeats and cross-reactivity with HRP3 when this gene is present. In the discordant samples, we found five samples with a low/non-predicted sensitivity, which agrees with the false-negative results in RDTs regardless the presence of *pfhrp3* gene. In contrast, we observed nine sensitive predicted samples lacking the *pfhrp3* gene. This false-negative results in RDT could be explained by a low parasitemia level (below 1,000 parasites/µL) and the absence of HRP3 as Kong et al. mentioned.^34^ However, for the sample with the highest parasitemia (14,414 parasites/µL) we consider that human error during the performance of the test occurred.

We also sequenced 6.95% (23/331) of the concordant samples and after applying the same approach of type 2 x type 7 score, all of them turned out to be sensitive to RDTs with scores above 60 [Supplementary data (Table)] and a parasitemia level above 1,000 parasites/µL. This indicates that an increased number of type 2 and type 7 repeats is necessary for the recognition of HRP2 when RDTs are used.

According to previous studies conducted in Colombia that show the sensitivity of the RDTs is affected more by antigen concentration than by parasitemia,^36^ a lower frequency of the repeats of interest due to partial deletions of the *pfhrp2* gene combined with the absence of the *pfhrp3* gene in 15 of the 16 samples could explain the negative result in the RDTs, regardless of the level of parasitemia.

Other factors could influence the performance of RDTs including improper storage, prolonged exposure to hot or humid conditions, human error in the interpretation of the RDT test bands, and whether the result was read before or after the incubation period recommended by the manufacturer. In addition, errors may occur in the pre-analytical phase related to an insufficient amount of blood or buffer, inadequate processing conditions of the sample or poor transport of RDT cassettes to their destination.^16^ The supervision and training in the field could not be carried out as desired, because of the coronavirus disease 19 (COVID-19) pandemic, which limited the ability to conduct in-person follow-up. However, virtual training and continuous supervision, as well as ongoing oversight of Pan American Health Organization (PAHO) field personnel mitigated the impact of these limitations.

Despite the limitations of this study, the findings provide valuable information about the prevalence of *pfhrp2/pfhrp3* gene deletions among malaria falciparum patients with false-negative RDT results and show the need to continue monitoring these genes in Colombia.

The results of this study are important since they demonstrate the good performance of HRP2-based RDTs in the detection of malaria. Their use could be maximized in areas where microscopy is unavailable, in outbreak situations, to decongest health services and provide diagnosis when laboratories are closed, and to expand the diagnostic network with volunteer collaborators and community workers. Our findings support the use of RDTs as an effective tool for the diagnosis and timely treatment of malaria in endemic areas of the Colombian Pacific Coast, although an acceptable prevalence of false negatives was observed in the RDTs, which was not related to *pfhrp2*-negative parasites. Even though no total deletion of the *pfhrp2* gene was found, a partial deletion was detected in two samples, which produced a band size smaller than expected. The effect of this partial deletion should be analyzed in detail to determine whether this deletion might affect the performance of the RDTs, possibly due to a decreased production of the HRP2 protein, or the lack of some epitopes recognized by the monoclonal antibodies present in the RDT cassettes. *Pfhrp2* gene deletion and its effects on the performance of HRP2-based RDTs should be studied alongside with the *hrp3* gene, considering the emergence of populations with *hrp3* deletions in the Pacific region.

It is necessary to strengthen the surveillance of *pfhrp2/3* deletion in Colombia to improve the quality of malaria diagnosis conducted through rapid tests. An accurate diagnostics network is essential, as false-negative results can lead to a lack of treatment and serious consequences, including death. False-negative results can also negatively affect the credibility of health services within the community.
