# Supplementary material for: Prevalence of pfhrp2/pfhrp3 gene deletions among patients with Plasmodium falciparum malaria with false-negative in the HRP2-based rapid diagnostic test in Colombia
Source: Mem Inst Oswaldo Cruz. 2025 Jun 13;120:e240134. doi: 10.1590/0074-02760240134 (PMC12165712; doi:10.1590/0074-02760240134)
Supplement: Supplementary file 1 [file 1678-8060-mioc-120-e240134-s.pdf]

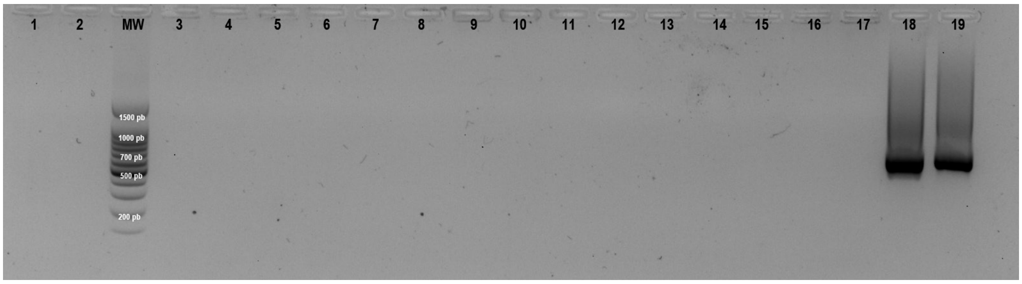

Agarose gel electrophoresis displaying the semi-nested polymerase chain reaction (PCR) products of the amplification of *pfhrp3* exon 2. Lanes 1 and 2 correspond to negative controls with water and DNA from Haiti strain lacking the *pfhrp3* gene respectively. Lanes 3 to 18 are the clinical samples showing a PCR amplicon only in GA12 (Lane 18). DNA from 3D7 strain was used as amplification control (Lane19). MW: 100 bp DNA Ladder (Promega). In the marker lane, the bands corresponding to 1500, 1000, 700, 500 and 200 bp are showed.

TABLE

Categorization of sensitivity to rapid diagnostic test (RDT) based on the type 2 (AHHAHHAAD) x type 7 (AHHAAD) HRP2 repeats present in concordant samples

Sample	Type 2	Type 7	Score	Category[a]	Sensitivity	Parasitemia	*pfHRP3* gene
B3	14	6	84	B	Sensitive	5,280	Absent
B4	12	7	84	B	Sensitive	3,680	Present
B7	11	9	99	B	Sensitive	8,760	Absent
B8	10	8	80	B	Sensitive	1,480	Absent
B20	11	7	77	B	Sensitive	1,418	Absent
GA01	10	7	70	B	Sensitive	5,440	Absent
GA03	10	8	80	B	Sensitive	18,920	Absent
GA04	12	6	72	B	Sensitive	6,560	Present
GA05	12	8	96	B	Sensitive	9,400	Absent
GA06	10	7	70	B	Sensitive	1,040	Absent
GA30	12	6	72	B	Sensitive	1,560	Absent
GE01	12	6	72	B	Sensitive	2,580	Absent
GE02	12	5	60	B	Sensitive	1,920	Absent
GE13	7	9	63	B	Sensitive	*	Absent
GE15	12	8	96	B	Sensitive	*	Present
TD08	12	8	96	B	Sensitive	1,800	Absent
TD21	11	9	99	B	Sensitive	1,072	Absent
TD22	10	8	80	B	Sensitive	6,720	Absent
TD23	10	7	70	B	Sensitive	5,160	Absent
TD24	10	9	90	B	Sensitive	33,613	Absent
TD26	10	8	80	B	Sensitive	4,720	Absent
TD27	12	7	84	B	Sensitive	10,040	Absent
TD31	14	8	112	A	Very sensitive	32,258	Absent
3D7[b]	11	5	55	B	Sensitive	-	Present

a: the categories shown are type A (score > 100, "very sensitive"), type B (score 50-99; "sensitive") or type C (< 50, "low/no sensitivity); *b*: reference strain. *The microscopy analysis for these samples couldn't be performed due to the preparation of the slides.

CC BY
